# Cabazitaxel Induced Thrombotic Microangiopathy in a Patient with Prostate Cancer

**DOI:** 10.1155/2019/8591283

**Published:** 2019-11-19

**Authors:** Yannick Nlandu, Franck Pourcine, Ly van phach Vong, Jonathan Chelly, Sebastien Jochmans, Sandie Mazerand, Mehran Monchi

**Affiliations:** Groupe Hospitalier Sud Ile-De-France, Centre Hospitalier de Melun, 77000 Melun, France

## Abstract

Cancer-associated thrombotic microangiopathy (TMA) refers to a group of disorders characterized by microangiopathic haemolytic anemia, thrombocytopenia, and ischemic organ damage. TMA manifestations can be induced by cancer or by chemotherapy. We report the case of a 64-year-old man with metastatic prostate cancer who experienced a Cabazitaxel-induced TMA manifestation. TMA responds to conservative therapy, dialysis without plasmaphoresis, with progressive recovered renal function.

## 1. Introduction

Thrombotic microangiopathy (TMA) is a clinical association of thrombocytopenia, microangiopathic haemolytic anaemia and organ dysfunction related to microvascular thrombosis [1, 2]. TMA is associated with significant mortality and morbidity including acute renal failure [3], a common feature caused by high susceptibility of renal vessels to endothelial damage [3]. In the course of cancer, TMA can be secondary to malignancy or to direct chemotherapy toxicity [4]. Al-Nouri et al. have established a list of anti-cancer agents associated with TMA with evidence of definitive causality, including a taxane drug, Docetaxel [5]. We report a case of TMA induced by Cabazitaxel, a newly integrated taxane drug in the treatment of advanced prostate cancer.

## 2. Case Report

**Figure 1:**
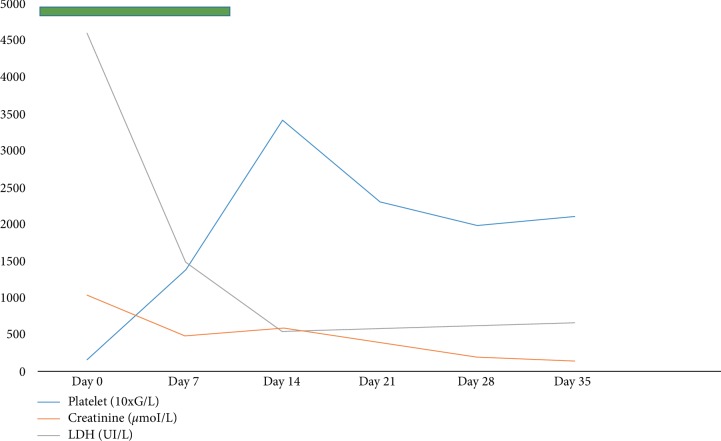
Laboratory parameters. Treatment period by haemodialysis is indicated by a coloured green bar. Day 0 represents TMA manifestations.

A 64-year-old male, is treated since 2005 for metastatic (spine and sub-diaphragmatic lymph nodes) and hormone-resistant prostate cancer. The patient was previously treated with Docetaxel and currently with Cabazitaxel every 3 weeks since 13^th^ june 2018. The level of Prostate-specific antigen (PSA) in March 2019 was 25.00 ng/mL for free PSA, and 898 ng/mL for total PSA. The patient who had previously received 18 cycles of Cabazitaxel at 25 mg/m^2^ (50 mg) and blood samples on 28^th^January 2019 showed a thrombocytopenia at 82 G/L and an elevated creatinine up to 328 *μ*mol/L with spontaneous normalization of creatinine and platelet count 7 weeks later after its discontinuation (Hb 9 g/dL, platelets 439G/L, creatinine 77 *μ*mol/L). Another injection of Cabazitaxel at lower dose (40 mg) was given on 21^th^ march 2019, with appearance 24 hours later of oliguria and asthenia. The file chart doesn't mention acute kidney injury (AKI) at the moment of reinjection. The clinical examination was unremarkable except for moderate hypertension at 150/90 mmHg. Blood samples on 26^th^ march showed an anemia with haemoglobin at 5 g/dL, thrombocytopenia at 17 G/L, schizocytes at more than 15% on blood smear, LDH at 4597 IU/L, haptoglobin <0.1 g/L, and normal INR at 1.14. The Coombs test was negative and there was renal insufficiency with creatinine at 1022 *μ*mol/L, urea 62 mmol /L. The profile of this renal failure was parenchymal with urinary sodium-potassium ratio >1. An ultrasound ruled out a post-renal AKI. The serum potassium was 5.7 mmol/L. Etiological investigations of this thrombotic microangiopathy showed 75% ADAMST13 activity, C4 at 0.45 g/L (0.10–0.40), C3 at 1.60 g/L (0.9–1.8), CH50 141% (70–130), 137% of factor H (65–140) and 180% factor I (60–130). The anti-factor H antibody was negative. The TMA aetiology looks as if associated with Cabazitaxel and chronologically compatible. Simultaneously to drug interruption, the patient had six dialysis sessions without plasmaphoresis. The evolution was characterized by progressive disappearance of biological signs of haemolysis and improvement of renal function (Figure 1). On May 3, 2019, haemoglobin level was 8.6 g/dL without schizocytes, platelets count at 209 G/L, creatinine at 137 *μ*mol/L, urea at 9 mmol/L, LDH at 661 IU/L and haptoglobin at 2.5 g/L. In this context, renal biopsy was not performed. The patient survived to the AKI KDIGO 3 episode with creatinine at 95 *μ*mol/L (eGFR 73 ml/min/1.73 m^2^) at 6 month.

## 3. Discussion

In a cancer patient, it is often difficult to distinguish chemotherapy-induced TMA from TMA caused by cancer [6]. Tumor-related TMA occurs in poorly controlled carcinomas, whereas chemotherapy-associated TMA is more common in disease remission or in minimal tumoral burden [7]. TMA induced by chemotherapy occurs by two main mechanisms: an immune-mediated reaction involving the development of drug-dependent antibodies or a direct endothelial damage [5, 6]. Direct toxicity is dose dependent in case of acute toxicity [5, 8]. The main anticancer agents associated with TMA are bleomycin, and mitomycin C [9, 10, 11]. In a systematic review, AL-Nouri et al. reported 22 drugs that had evidences for association with TMA, including Docetaxel, a taxane class molecule [5].They defined the typical clinical manifestations of an immune reaction by the onset of symptoms within 21 days for a drug administered daily or in the hours following exposure (within 24 hours) for a drug taken intermittently [5]. Direct toxicity was defined either by the acute onset of symptoms after exposure to the offending drug or by the progressive development of toxicity in the form of acute renal failure [5]. Cabazitaxel is used for prostatic cancer [12] in combination with prednisolone. Renal insufficiency following treatment with Cabazitaxel is very rare, but cases of nephrotoxicity in patients have been reported [13]. Most of the cases of renal insufficiency described are associated with sepsis, dehydration or obstructive uropathy, without evidence for a direct nephrotoxicity [13]. Tumma et al. reported a case of TMA, 48 hours after the first dose of Cabazitaxel, with a fatal renal failure [7]. In our case, the occurrence after 18 cycles of Cabazitaxel and the regression of TMA after withdrawal of the treatment suggests a cumulative dose nephrotoxicity of this new treatment. The taxanes have significant antiangiogenic properties in vitro and in vivo [14]. They inhibits endothelial function and angiogenesis and shows a dose-dependent vascular toxicity [14]. In this context, taxane can impair renal vasculature and cause TMA. Our patient had already received 18 injections of Cabazitaxel with a first episode suggesting TMA in January 2019 (platelets at 82 G/L and creatinine at 328 *μ*mol/L with normalization in February, at distance from injections). The current episode was following an injection on march 21, 2019 (haemoglobin 9 g/dL, platelet 439 G/L, creatinine 77 *μ*mol/L before the injection).

## 4. Conclusion

We report a case of thrombotic microangiopathy due to Cabazitaxel, a toxoid used in the treatment of advanced prostate cancer.
